# Predictive value of post-procedural early (within 24 h) increase in cystatin C for contrast-induced acute kidney injury and mortality following coronary angiography or intervention

**DOI:** 10.18632/oncotarget.19034

**Published:** 2017-07-06

**Authors:** Yong Liu, Kai-Hong Chen, Shi-Qun Chen, Li-Ling Chen, Chong-Yang Duan, Kun Wang, Xiao-Sheng Guo, Hua-Long Li, Wei-Jie Bei, Kai-Yan Lin, Ping-Yan Chen, Ying Xian, Ning Tan, Ying-Ling Zhou, Qing-Shan Geng, Ji-Yan Chen

**Affiliations:** ^1^ Department of Cardiology, Guangdong Cardiovascular Institute, Guangdong Key Laboratory of Coronary Disease Prevention, Guangdong General Hospital, Guangdong Academy of Medical Sciences, School of Medicine, South China University of Technology, Guangzhou, China; ^2^ Department of Cardiology, Longyan First Affiliated Hospital of Fujian Medical University, Longyan, Fujian Province, China; ^3^ Department of Cardiology, Guangdong General Hospital Zhuhai Hospital, Zhuhai, Guangdong, China; ^4^ National Clinical Research Center for Kidney Disease, State Key Laboratory of Organ Failure Research, Department of Biostatistics, School of Public Health and Tropical Medicine, Southern Medical University, Guangzhou, China; ^5^ Southern Medical University, Guangzhou, Guangdong, China; ^6^ Duke Clinical Research Institute, Durham, NC, USA

**Keywords:** cystatin C, coronary angiography, percutaneous coronary intervention, contrast-induced acute kidney injury

## Abstract

**Objective:**

To investigate the predictive value of post-procedural early (within 24 h) increase in cystatin C for contrast-induced acute kidney injury (CI-AKI) and all-cause mortality following coronary angiography or intervention.

**Methods:**

We prospectively investigated 1042 consecutive patients with both baseline and early post-procedural cystatin C measurement undergoing coronary angiography or intervention. CI-AKI was defined as an increase ≥0.3 mg/dL or >50% in serum creatinine from baseline within 48 h post-procedure. Mean follow-up was 2.26 years.

**Results:**

Overall, the patients had a CI-AKI incidence was 3.6% (38/1042), mean serum creatinine of 87 µmol/L. Compared with Mehran risk score, post-procedural early absolute increase (AUC: 0.584 *vs*. 0.706, *P* = 0.060) and relative increase (AUC: 0.585 *vs*. 0.706, *P* = 0.058) in cystatin C had poorer predictive value for CI-AKI. According to multivariate analysis, post-procedural significant early increase (≥0.3 mg/dL or ≥10%) in cystatin C developed in 231 patients (22.2%), was not independent predictor of CI-AKI (adjusted OR: 1.23, 95% CI, 0.56–2.69, *P* = 0.612), and long-term mortality (adjusted HR: 0.90; *P* = 0.838).

**Conclusions:**

Our data suggested post-procedural early increase (within 24 h) in cystatin C was not effective for predicting CI-AKI or all-cause mortality following coronary angiography or intervention among patients at relative low risk of CI-AKI, the negative finding of poor predictive value should be further evaluated in larger multicenter trials.

## INTRODUCTION

Contrast-induced acute kidney injury (CI-AKI) is a common complication after percutaneous coronary intervention (PCI) and is associated with prolonged hospital stay, increased medical costs, and increased risk of adverse clinical outcomes [1, 2]. Since effective treatment measures for CI-AKI have not been established and the treatment window is narrow, early identification of patients at high risk of CI-AKI is important [3].

Serum creatinine (SCr) is a common index used to evaluate renal function and define CI-AKI. However, SCr concentration is affected by muscle mass, age, sex, and diet, and may not change until a significant loss of renal function [4-6]. Therefore, SCr concentration could be unreliable and overestimate the estimated glomerular filtration rate (eGFR).

Cystatin C, another biomarker to evaluate renal function, is produced at a constant rate by nucleated cells and reabsorbed in the proximal renal tubules [7]. Compared with SCr, the concentration of cystatin C is less affected by muscle mass, age, sex, and diet, and its half-life is 3 times shorter [8, 9], allowing for the reliable and early detection of changes in cystatin C levels in blood. Some previous studies indicated that the relative increase of cystatin C is a sensitive early biomarker of CI-AKI after contrast exposure [10, 11]. By contrast, other studies reported a low predictive value of CI-AKI [12, 13]. Previous studies have focused on specific patient groups (e.g. chronic kidney disease), but the association of cystatin C with CI-AKI in more general patient populations remains unknown. Therefore, the purpose of our study was to analyze the association of post-procedural early (within 24 h) increase in cystatin C with CI-AKI and long-term all-cause mortality following coronary angiography or intervention.

## RESULTS

### Clinical characteristics

**Figure 1:**
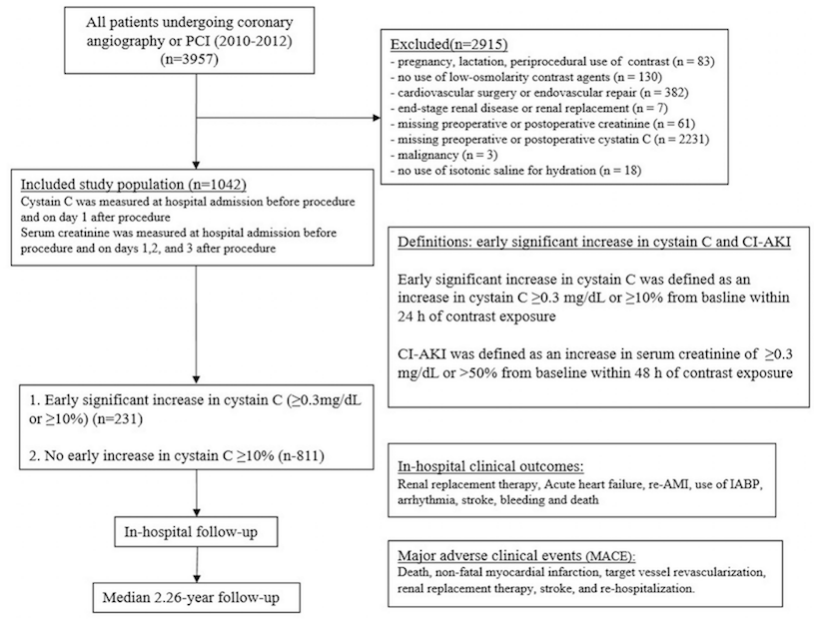
Flow diagram of the selection process

A total of 1042 patients were included in the final analysis (mean age 62.87 ± 10.35 years, mean creatinine clearance rate 75.12 ± 26.24 mL/min, and mean cystatin C 1.20 ± 0.47 mg/L), and 297(28.5%) patients presented with pre-existing chronic kidney disease (CKD), creatinine clearance <60 ml/min. The protocol and flow diagram of the selection process is presented in Figure 1.

Patients’ baseline characteristics are listed in Table 1 and Table 2. Overall, 38 patients (3.6%) developed CI-AKI. Compared to patients without CI-AKI, patients with CI-AKI were older. Furthermore, those in the CI-AKI group had a lower creatinine clearance rate and higher Mehran risk score (Table 1).

**Table 1:** Characteristics of patients with or without CI-AKI

Characteristic	All patients	CI-AKI	Non-CI-AKI	*P* value
	*n* = 1042	*n* = 38	*n* = 1004	
Age	62.87±10.35	68.08±10.05	62.67±10.31	0.002
Sex (male)	784 (75.2%)	23 (60.5%)	761 (75.8%)	0.032
Serum creatinine, µmol/L				
Baseline	87.22±38.23	97.92±45.57	86.82±37.89	0.079
Within 24 h post-procedure	88.82±34.57	138.28±56.17	86.94±32.05	<0.001
Within 48 h post-procedure	93.76 ± 46.57	130.72±67.82	90.70±43.09	0.005
Within 72 h post-procedure	105.23 ± 95.94	149.73±146.97	100.12±88.66	0.413
Serum cystatin C, mg/L				
Baseline	1.20 ± 0.47	1.56±0.60	1.18±0.46	<0.001
Within 24 h post-procedure	1.16±0.49	1.65±0.62	1.14±0.48	<0.001
Within 48 h post-procedure	1.20 ± 0.51	1.51±0.63	1.18±0.49	0.002
Creatinine clearance rate, ml/min	75.12±26.24	67.27±38.28	75.42±25.65	0.201
>90	242 (23.3%)	6 (15.8%)	236 (23.6%)	<0.001
60-90	501 (48.2%)	10 (26.3%)	491 (49.0%)	
30-60	274 (26.3%)	20 (52.6%)	254 (25.3%)	
<30	23 (2.2%)	2 (5.3%)	21 (2.1%)	
Mehran risk score	4.54±3.60	8.20±5.50	4.41±3.45	<0.001
£5	778 (77.0%)	15 (42.9%)	763 (78.2%)	<0.001
6-10	176 (17.4%)	10 (28.6%)	166 (17.0%)	
11-15	41 (4.1%)	4 (11.4%)	37 (3.8%)	
≥16	16 (1.6%)	6 (17.1%)	10 (1.0%)	
Δcystatin C ≥0.3 mg/L within 24 h	76 (7.3%)	6 (15.8%)	70 (7.0%)	0.040
Δcystatin C ≥10% within 24 h	231 (22.2%)	9 (23.7%)	222 (22.1%)	0.819
Δcystatin C ≥0.3 mg/L or ≥10% within 24 h	231 (22.2%)	9 (23.7%)	222 (96.1%)	0.819
Δcystatin C ≥0.5 mg/L within 24 h	28 (2.7%)	5 (13.2%)	23 (2.3%)	<0.001
Δcystatin C ≥25% within 24 h	97 (9.3%)	7 (18.4%)	90 (9.0%)	0.049
Δcystatin C ≥0.5 mg/L or ≥25% within 24 h	97 (9.3%)	7 (18.4%)	90 (9.0%)	0.049

Abbreviations: CI-AKI=contrast-induced acute kidney injury.

**Table 2:** Characteristics of patients with or without significant Δcystatin C

Variables	All patients	Significant Δcystatin C	Non-significant Δcystatin C	*P* value
	*n* = 1042	*n* = 231	*n* = 811	
**Demographic variables**				
Age, years	62.87±10.35	61.26±10.51	63.33±10.26	0.007
Age >75 years	116 (11.1)	17 (7.4)	99 (12.2)	0.039
Men	784 (75.2)	171 (74.0)	613 (75.6)	0.628
Weight, kg	65.70±11.02	65.61±10.98	65.98±11.20	0.654
Smokers	389 (37.3)	94 (40.7)	295 (36.4)	0.255
Hypertension	608 (58.3)	127 (55.0)	481 (59.3)	0.239
Diabetes mellitus	260 (25.0)	62 (26.8)	198 (24.5)	0.464
Anemia	365 (35.6)	67 (29.6)	298 (37.3)	0.034
Dyslipidemia	157 (15.1)	32 (13.9)	125 (15.4)	0.559
Congestive heart failure	119 (11.5)	18 (8.0)	101 (12.5)	0.057
Previous MI	125 (12.0)	30 (13.0)	95 (11.7)	0.599
Previous CABG	11 (1.1)	3 (1.3)	8 (1.0)	0.682
**Biochemical parameters**				
SBP, mmHg	129.81 ±18.90	130.39±20.49	129.64±18.43	0.616
Hypotension	14 (1.3)	5 (2.2)	9 (1.1)	0.221
LVEF	59.89±12.67	59.94±12.78	59.69±12.33	0.804
LVEF <40%	82 (9.7)	19 (9.9)	63 (9.6)	0.902
Baseline SCr, μmol/L	87.22±38.23	84.81±29.22	87.91±40.42	0.195
Baseline CrCl, mL/min	75.12±26.24	78.34±26.14	74.20±26.21	0.034
HbA1c, %	6.51±1.27	6.51±1.17	6.51±1.30	0.949
Hemoglobin, g/dL	133.87±15.40	134.53±16.61	133.69±15.04	0.466
Total cholesterol, mmol/L	4.38 ± 1.11	4.45±1.21	4.37 ± 1.08	0.359
HDL-C, mmol/L	0.93±0.31	0.93 ± 0.33	0.93±0.30	0.992
LDL-C, mmol/L	2.56±0.92	2.62 ± 1.08	2.54 ± 0.87	0.311
Mehran risk score	4.54±3.60	4.07 ± 3.43	4.67 ± 3.64	0.027
**Medication Therapy**				
Diuretic	132 (12.7)	29 (12.6)	103 (12.7)	0.953
ACEI/ARB	923 (88.6)	203 (87.9)	720 (88.8)	0.704
β-Blockers	942 (90.4)	205 (88.7)	736 (90.9)	0.332
Calcium channel blockers	186 (17.9)	47 (20.3)	139 (17.2)	0.268
**Procedure performed**				
Coronary lesion	1.88±1.18	1.81±1.19	1.90±1.17	0.287
Emergency PCI	16 (1.5%)	4 (1.7%)	12 (1.5%)	0.784
IABP	7 (0.7%)	1 (0.4%)	6 (0.7%)	0.614
No. of stent used	1.41 ± 1.25	1.37±1.20	1.42±1.27	0.675
Total stent length, mm	34.92±33.59	34.62±32.30	35.01±33.97	0.884
Procedure duration, min	64.54±45.83	63.56±46.75	64.82±45.59	0.718
Contrast volume, mL	117.98±64.80	117.90±66.40	118.01±64.38	0.982
Hydration volume, mL	697.16±370.88	680.80±355.58	701.85±375.23	0.447

Abbreviations: ACEI/ARB = angiotensin converting enzyme inhibitor/angiotensin receptor blocker; CABG = coronary artery bypass grafting; CrCl = creatinine clearance ; eGFR = estimated glomerular clearance rate; HbA1c = hemoglobin A1c; HDL-C = high-density lipoprotein cholesterol; IABP = intra-aortic balloon pump; LDL-C = low-density lipoprotein cholesterol; LVEF = left ventricular ejection fraction; MI = myocardial infarction; PCI = percutaneous coronary intervention; SBP = systolic blood pressure; SCr = serum creatinine.

In addition, 231 patients (22.2%) demonstrated a significant Δcystatin C. These patients were also more likely to be older and to have renal insufficiency, anemia, and higher Mehran risk score. However, the medication therapy, procedure process, and other demographic characteristics and biochemical parameters were similar between the two groups (Table 2). The incidence of CI-AKI did not differ between patients with and without a significant Δcystatin C (3.9% vs. 3.6%, respectively; P=0.819). Similar results were found in other in-hospital outcomes (Table 3).

**Table 3:** In-hospital outcomes of the two groups

	All patients	Significant Δcystatin C (≥0.3 mg/dL or ≥10%)	Non-significant Δcystatin C	*P* value
CI-AKI	38 (3.6)	9 (3.9)	29 (3.6)	0.819
Mortality	2 (0.2)	0 (0.0)	2 (0.2)	0.450
AHF	7 (0.7)	0 (0.0)	7 (0.9)	0.155
RRT	4 (0.4)	0 (0.0)	4 (0.5)	0.284
Re-AMI	1 (0.1)	0 (0.0)	1 (0.1)	0.593
Arrhythmia	7 (0.7)	1 (0.4)	6 (0.7)	0.614
Stroke	16 (1.5)	5 (2.2)	11 (1.4)	0.379
Bleeding	4 (0.4)	2 (0.9)	2 (0.2)	0.180

Abbreviations: AHF= acute heart failure; CI-AKI = contrast-induced acute kidney injury; RRT = renal replacement therapy; Re-AMI = recurrent acute myocardial infarction; IABP = intra-aortic balloon pump.

### Association between cystatin C and CI-AKI

**Figure 2:**
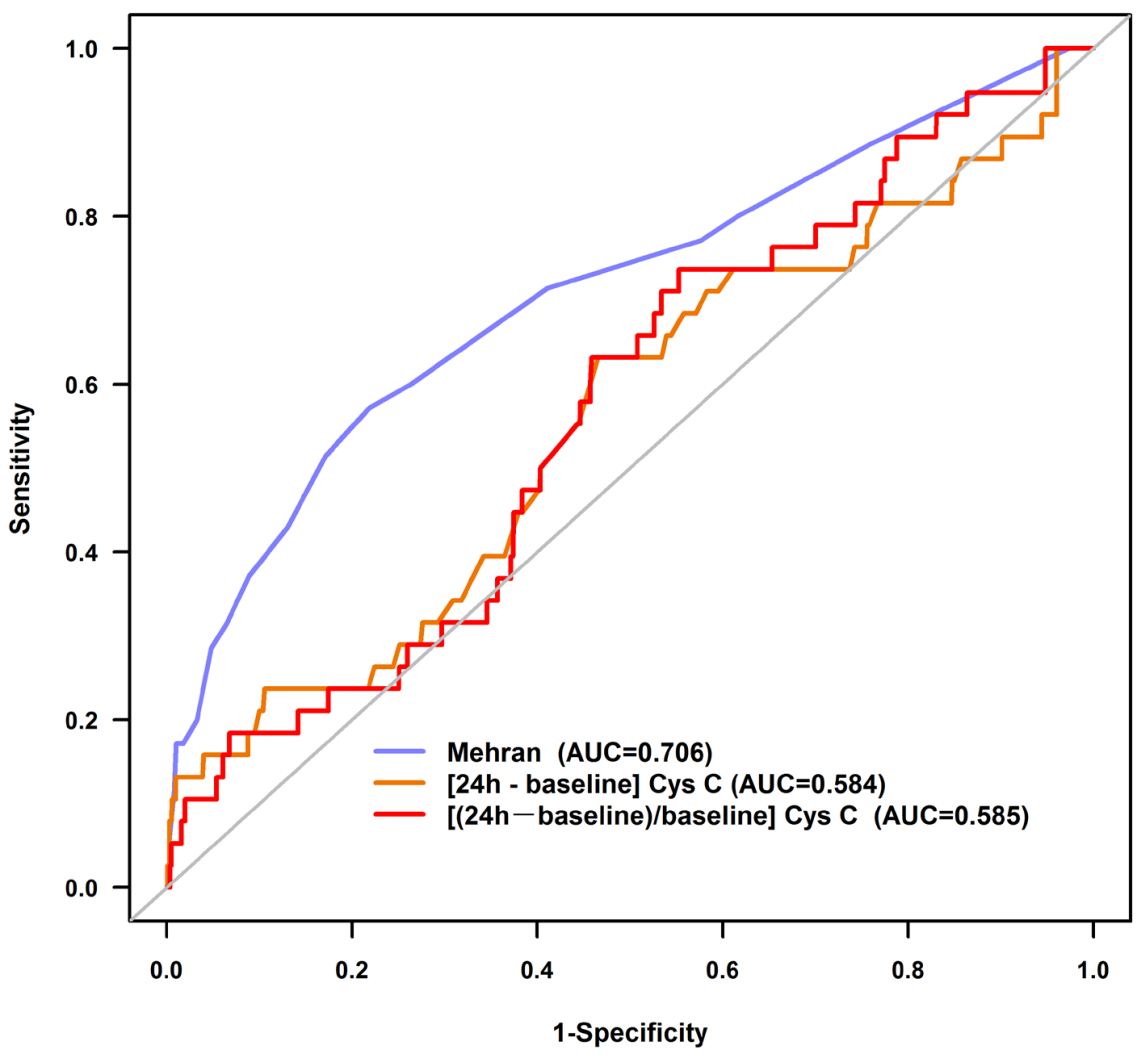
Receiver operating characteristic curves for contrast-induced acute kidney injury

ROC curve analysis revealed that early absolute or relative increase in cystatin C (as Δcystatin C, within 24 h) had poorer predictive value than Mehran risk score for CI-AKI (AUC=0.584 vs. 0.706, P=0.060; AUC=0.585 vs. 0.706, P=0.058; respectively) (Figure 2). We added Cystatin C in Mehran risk model, and the result shows that Cystatin C can’t significantly improve the prediction value (Supplementary Figure 1).

After controlling for other confounders, including age >75 years, creatinine clearance <60 mL/min, emergency PCI, intra-aortic balloon pump, male, weight>60kg, smoke, C-reactive protein (CRP)>3mmol/l and hydration volume ≤960 mL, multivariate logistic regression results revealed that significant Δcystatin C (≥0.3 mg/dL or ≥10%) was not associated with CI-AKI (OR=0.88, 95%CI, 0.32-2.41, P=0.798), so did Δcystatin C absolute increase ≥0.5 mg/L or relative increase≥25% (OR=2.15, 95%CI, 0.68∼6.81, P=0.195) (Table 4).

**Table 4:** Multivariate analyses for predictors of contrast-induced nephropathy (CIN)

Variables	Adjusted OR	95% CI	*P* value
ΔCystatin C >0.3 mg/L to predict CIN	1.82	0.50–6.62	0.362
ΔCystatin C >10% to predict CIN	0.88	0.32–2.41	0.798
ΔCystatin C >0.3 mg/L or 10% to predict CIN	0.88	0.32–2.41	0.798
ΔCystatin C >0.5 mg/L to predict CIN	5.28	1.36∼20.57	0.016
ΔCystatin C >25% to predict CIN	2.15	0.68∼6.81	0.195
ΔCystatin C >0.5 mg/L or 25% to predict CIN	2.15	0.68∼6.81	0.195

CIN was adjusted for age >75 years, creatinine clearance <60 mL/min, emergency percutaneous coronary intervention, intra-aortic balloon pump, male sex, weight>60kg, smoking, C-reactive protein >3mmol/L, and hydration volume ≤960 mL.

### Association of cystatin C with long-term outcomes

**Figure 3:**
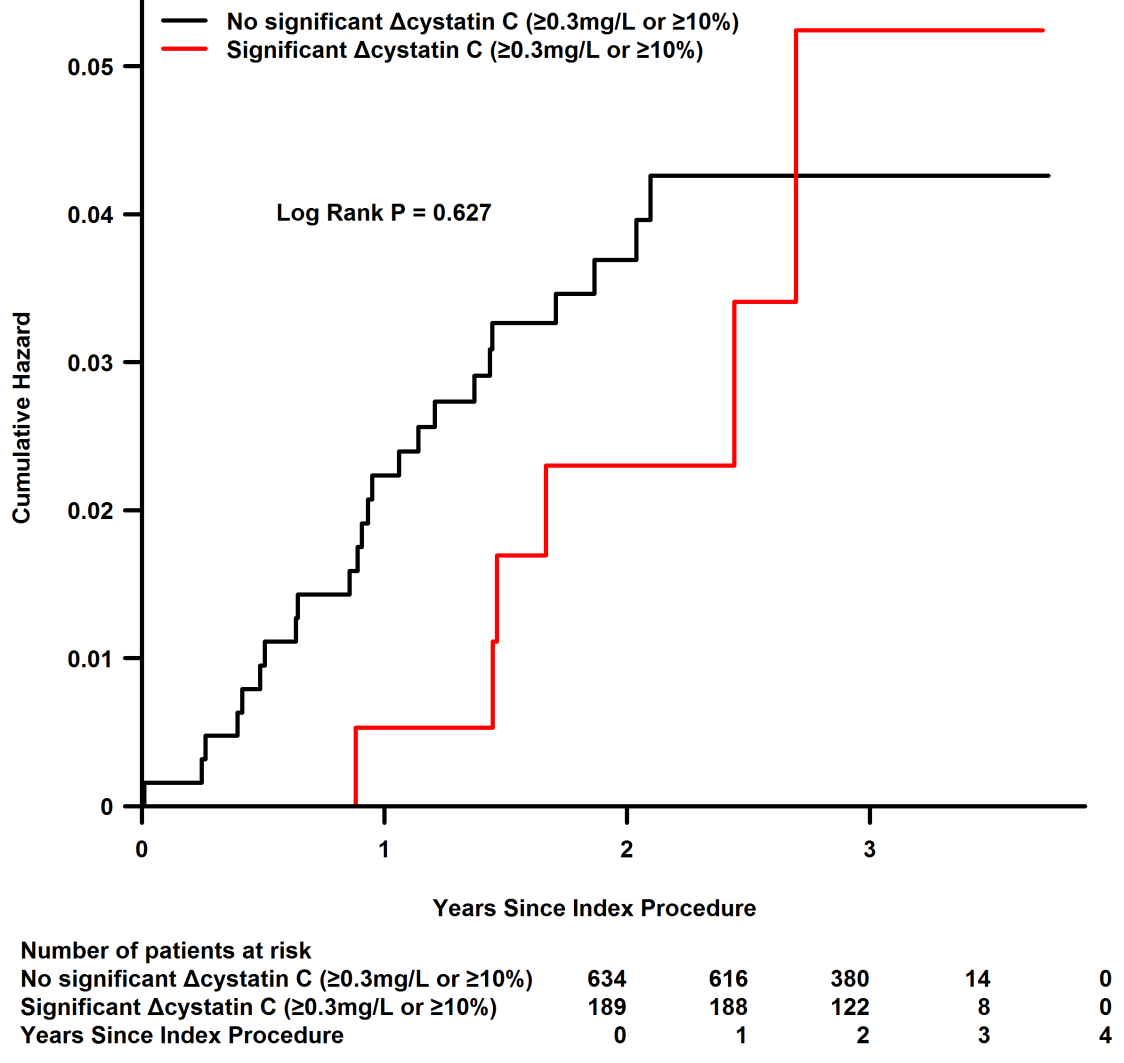
Kaplan-Meier curve for long-term mortality

**Figure 4:**
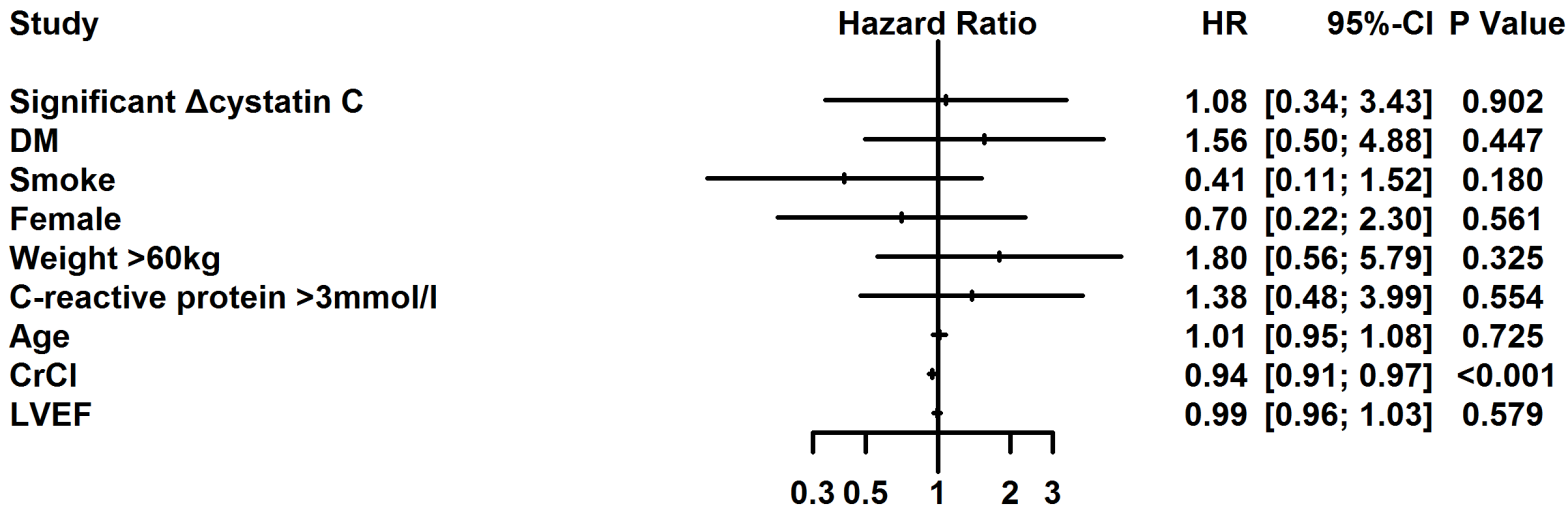
Multivariate Cox regression analysis for long-term mortality

The mean follow-up period was 2.26 years. Kaplan-Meier curve analysis revealed that significant Δcystatin C was not associated with increased long-term mortality (P=0.627) (Figure 3). After adjusting for confounders, including age, diabetes, creatinine clearance, female gender, weight>60kg, smoke, C-reactive protein (CRP) >3mmol/l and LVEF, significant Δcystatin C (≥0.3 mg/dL or ≥10%) was not an independent risk factor for long-term mortality (HR=1.08, 95%CI, 0.34-3.43, P=0.902) (Figure 4).

## DISCUSSION

To our knowledge, this study is the largest to date to investigate the relation of change in cystatin C to CI-AKI and long-term mortality in patients undergoing coronary angiography or intervention. Because CI-AKI is associated with renal and cardiovascular adverse events and long-term mortality, its early identification is critical. However, our data indicated that early significant increase in cystatin C did not have good predictive value for CI-AKI and long-term mortality.

CI-AKI is a common and serious complication after contrast exposure, which can affect up to 50% of patients with additional risk factors, such as administration of a high dose of CM, undergoing emergency PCI, and chronic kidney disease [3]. However, the incidence of CI-AKI in our analysis (3.6%) was lower than that in previous reports. Our study population was not limited to patients with acute coronary syndrome. Furthermore, some patients who underwent coronary angiography without stent implantation may have received a low dose of CM (30-50 ml) during the procedure. In addition, fewer than half of patients had chronic kidney disease. All of the abovementioned reasons may have contributed to the lower CI-AKI incidence observed in this study.

Cystatin C, a 13-kDa cysteine protease inhibitor, is produced at a constant rate by nucleated cells. Compared with SCr, cystatin C is reabsorbed and almost completely catabolized in the proximal renal tubules. Due to its low molecular weight and positive charge at physiological pH, the serum concentration of cystatin C is determined by glomerular filtration and less influenced by age, sex, muscle mass, and nutrition than SCr. In addition, cystatin C is only present in the extracellular space, leading to its quicker increase and shorter half-life in serum [8, 9]. Therefore, the concentration of cystatin C can rapidly increase after CM exposure, and it had been postulated to be valuable for the early prediction of CI-AKI.

Previous studies demonstrated that Δcystatin C was an independent predictor of CI-AKI. A study including a total of 204 patients undergoing primary angioplastyfound that cystatin C relative increase ≥10% within 72 h had a good predictive value for CI-AKI, with 96.30% sensitivity and 67.23% specificity [11]. Similarly, a recent prospective analysis further confirmed the value of Δcystatin C for CI-AKI in patients undergoing elective coronary angiography [14]. Furthermore, Kim et al demonstrated similar results in patients with peripheral artery disease after contrast exposure [10]. Although cystatin C rapidly peaks within 24 h after contrast exposure and decreases thereafter, all of these previous studies analyzed Δcystatin C within 48-72h, which might influence its predictive value. By contrast, an observational study by Ribichini et al including 166 patients who underwent coronary angioplasty analyzed the change in cystatin C within 24 h and indicated that Δcystatin C >10% within 24 h was not associated with an elevated risk of CI-AKI [12]. Furthermore, the early changes in cystatin C levels (12 h from baseline) were not superior to changes in SCr levels for predicting CI-AKI in patients who underwent coronary angioplasty. The variable definition of CI-AKI, differences in treatment or measurement time, and disparate patient populations may all have contributed to these conflicting results. To overcome these obstacles, the present study included a relatively large population of patients who underwent cardiac catheterization and analyzed the predictive value of Δcystatin C within 24 h for CI-AKI.

The increase in serum cystatin C levels after ST-elevation myocardial infarction could also be a predictor of medium-term major adverse cardiovascular events [15]. A meta-analysis of human studies demonstrated that patients with elevated cystatin C levels prior to the diagnosis of acute kidney injury have worse outcomes. However, the analysis failed to identify a consensus cut-off value to define cystatin C level elevation [16], potentially because cystatin C is not only a sensitive marker of kidney function but also associated with atherosclerosis and cardiac structural abnormalities. Furthermore, cystatin C has unforeseen toxic effects that also contribute to the strength of its association with mortality and cardiovascular risk [17]. By contrast, we found that an early increase in cystatin C (relative or absolute 24-h post-procedural change) did not predict CI-AKI or long-term adverse outcomes. Additional comprehensive studies with larger samples are needed to determine whether early changes in cystatin C levels are a better predictor for CI-AKI and major adverse cardiovascular events.

We acknowledge several limitations of our study. First, this prospective observational study was conducted at a single center with a limited sample size, which prevents these findings from being extended to other patient populations until they are confirmed by larger multicenter clinical trials. Second, variation of post-procedural cystatin C measurement times may have led to peak levels of cystatin C being missed. Third, the high missing data rate (70%) of systemic cystatin C measurement (baseline or post-procedural) increased the bias of the population selection, which further limits the extension of our findings. Fourth, approximately half of patients were discharged at 48 h after coronary angiography or interventions, which may have led to an underestimation of the true incidence of CI-AKI. Fifth, the incidence of CI-AKI, which served as the primary end-point, was low among patients with low Mehran score and serum creatinine, and we did not have adequate data to investigate the contrast limit in high-risk patients. Sixthly, all the data of cystatin C did not come from urine sample, which may be more sensitive and early to detect CI-AKI, but come from blood sample. Finally, although we previously promoted the use of a hydration protocol, no strictly uniform protocol was applied in this observational study, which may have influenced the dynamic change of post-procedural cystatin C and the incidence of CI-AKI.

In conclusion, our data suggest that post-procedural early increase (within 24 h) in cystatin C is not effective for predicting CI-AKI or all-cause mortality following coronary angiography or intervention among patients at low risk of CI-AKI. The reverse finding of poor predictive value for post-procedural early increase in cystatin C needs further evaluation in larger multicenter trials.

## MATERIALS AND METHODS

### Study population

This study included 3957 consecutive patients more than 18 years old who underwent coronary angiography or intervention according to the institutional protocol between January 2010 and October 2012 at Guangdong General Hospital. In accordance with the updated European guidelines on contrast media [3], the exclusion criteria included pregnancy, lactation, intravascular administration of a contrast medium (CM) within the previous 7 days or 3 days postoperation, no use of low-osmolality contrast agents, cardiovascular surgery or endovascular repair, end-stage renal disease or renal replacement, missing preoperative or postoperative creatinine, missing preoperative or postoperative cystatin C, malignancy, and no use of isotonic saline for hydration. Consequently, 1042 patients with both preoperative baseline and early postoperative (<24 h) measurement of cystatin C were included in the final study (Figure 1).

This study was performed according to the Declaration of Helsinki, and the ethics committee of the Guangdong General Hospital approved the study protocol. Written informed consent was obtained from the patients involved in the study.

### Biochemical investigations

SCr concentration was measured at admission and within 24 h, 48 h, and 72 h after CM administration. Cystatin C was measured prior to the procedure and within 24 h after the procedure. Other biochemical indicators, such as hemoglobin (Hb) A1c, lipid profile, and Hb level, were measured in the morning prior to the procedure. The Cockcroft-Gault formula was used to calculate the eGFR [18].

### Cardiac catheterization

The cardiac catheterization procedure was performed via a femoral or radial approach by experienced interventional cardiologists according to standard clinical practice. Non-ionic, low-osmolality CM was used for all patients (either Iopamiron^®^ or Ultravist^®^, both 370 mg I/mL). Patients received a continuous intravenous infusion of isotonic saline at a rate of 1 mL/kg/h (0.5 mL/kg/h in cases of left ventricular ejection fraction <40% or severe congestive heart failure) for at least 2-12 h before and 6-24 h after the procedure. The use of drugs was based on the patient’s condition and PCI guidelines [19].

### Clinical end-points and follow-up

The primary end-point of this study was the development of CI-AKI, defined as an absolute increase ≥0.3 mg/dL or a relative increase >50% from baseline SCr level within 48 h after contrast exposure [20]. Additional end-points included significant early absolute or relative increase in cystatin C (Δcystatin C), defined as an absolute increase ≥0.3 mg/dL or relative increase ≥10% from baseline within 24 h after contrast exposure, and all-cause mortality [10].

During the mean follow-up period of 2.26 years (interquartile range, 1.84-2.68 years), events were carefully monitored and recorded by trained nurses through office or telephone visits at 1, 6, 12, 24, 36, and 48 months after discharge.

### Statistical analysis

For continuous variables, t-tests were used for normally distributed data (described as mean ± standard deviation), and the Wilcoxon rank-sum test was used for data with non-normal distribution (described as interquartile range). For categorical variables, χ2 test or Fisher’s exact test was used (described as absolute values and percentages). Receiver operating characteristic (ROC) curve analysis was conducted to assess the predictive value of Δcystatin C for CI-AKI. Area under the curve (AUC) values were compared between Δcystatin C and Mehran score by using MedCalc statistical software (MedCalc software, version 11.4, Mariakerke, Belgium) [21]. Multivariate logistic regression and Cox proportional hazards regression analyses were performed to identify the independent risk factors for CI-AKI and long-term mortality, respectively. The Kaplan-Meier method was used to describe the all-cause mortality by log-rank tests. All data analyses were performed using SAS version 9.4 (SAS Institute, Cary, NC, USA). A two-tailed *P<*0.05 was considered statistically significant.

## SUPPLEMENTARY MATERIALS FIGURE


