# Correction: Predictive value of post-procedural early (within 24 h) increase in cystatin C for contrast-induced acute kidney injury and mortality following coronary angiography or intervention

**DOI:** 10.18632/oncotarget.24406

**Published:** 2017-02-05

**Authors:** Yong Liu, Kai-Hong Chen, Shi-Qun Chen, Li-Ling Chen, Chong-Yang Duan, Kun Wang, Xiao-Sheng Guo, Hua-Long Li, Wei-Jie Bei, Kai-Yan Lin, Ping-Yan Chen, Ying Xian, Ning Tan, Ying-Ling Zhou, Qing-Shan Geng, Ji-Yan Chen

**Affiliations:** ^1^ Department of Cardiology, Guangdong Cardiovascular Institute, Guangdong Key Laboratory of Coronary Disease Prevention, Guangdong General Hospital, Guangdong Academy of Medical Sciences, School of Medicine, South China University of Technology, Guangzhou, China; ^2^ Department of Cardiology, Longyan First Affiliated Hospital of Fujian Medical University, Longyan, Fujian Province, China; ^3^ Department of Cardiology, Guangdong General Hospital Zhuhai Hospital (Zhuhai Golden Bay Center Hospital), Zhuhai, Guangdong, China; ^4^ National Clinical Research Center for Kidney Disease, State Key Laboratory of Organ Failure Research, Department of Biostatistics, School of Public Health and Tropical Medicine, Southern Medical University, Guangzhou, China; ^5^ Southern Medical University, Guangzhou, Guangdong, China; ^6^ Duke Clinical Research Institute, Durham, NC, USA

**Previous name:**

^3^ Department of Cardiology, Guangdong General Hospital Zhuhai Hospital, Zhuhai, Guangdong, China

**Current name:**

^3^ Department of Cardiology, Guangdong General Hospital Zhuhai Hospital (Zhuhai Golden Bay Center Hospital), Zhuhai, Guangdong, China

Original article: Oncotarget. 2017; 8:109762-109771. https://doi.org/10.18632/oncotarget.19034

