# Supplementary material for: Predictive value of post-procedural early (within 24 h) increase in cystatin C for contrast-induced acute kidney injury and mortality following coronary angiography or intervention
Source: Oncotarget. 2017 Jul 6;8(65):109762–71. doi: 10.18632/oncotarget.19034 (PMC5752559; doi:10.18632/oncotarget.19034)
Supplement: Supplementary file 1 [file oncotarget-08-109762-s001.pdf]

Predictive value of post-procedural early (within 24 h) increase in cystatin C for contrast-induced acute kidney injury and mortality following coronary angiography or intervention

SUPPLEMENTARY MATERIALS

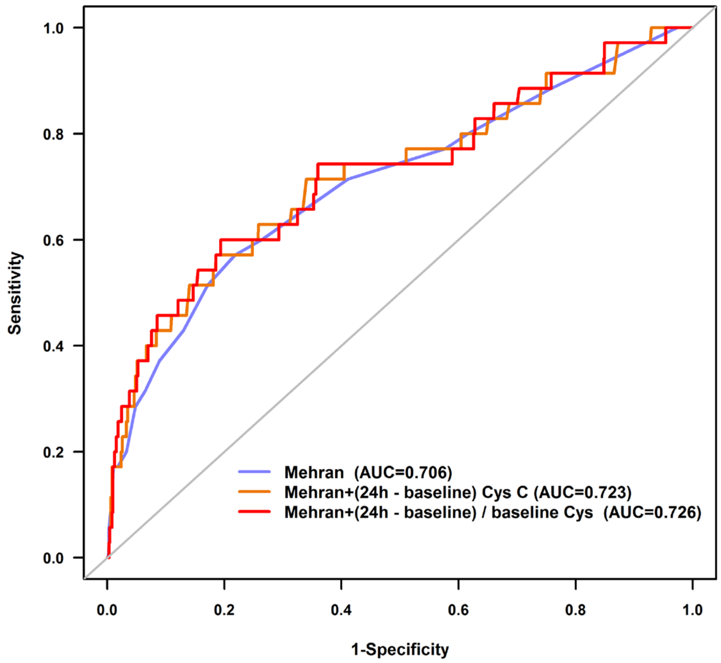

Supplementtray Figure 1: Predictive value of Mehran score after adding cystatin C
